# The impact of artistic sports on academic self-efficacy

**DOI:** 10.3389/fpsyg.2024.1458460

**Published:** 2025-01-22

**Authors:** Yanyan Tian, Haiqing Wang

**Affiliations:** ^1^Lanzhou Petrochemical University of Vocational Technology, Lanzhou, China; ^2^School of Psychology, Northwest Normal University, Lanzhou, China

**Keywords:** artistic sports, sports training types, five factor mindfulness, social anxiety, academic procrastination, academic self-efficacy

## Abstract

**Introduction:**

Artistic sports have a more positive impact on adolescents on the basis of basic sports. This study delves into the beneficial effects of Artistic sports compared to basic sports in enhancing academic self-efficacy in college students, and investigates the mediating roles of mindfulness, social anxiety, and academic procrastination in this process.

**Methods:**

A questionnaire survey was conducted among students in some universities in Gansu Province, collecting a total of 1,976 online questionnaires, including 263 males and 1,713 females, with 1,543 participants in Artistic sports courses and 433 participants in basic sports. Data processing was carried out using SPSS 26.0 software and its plugin PROCESS.

**Results:**

The analysis results indicate significant differences in mindfulness, social anxiety, academic procrastination, and academic self-efficacy among different types of sports training (*ps* < 0.05); significant correlations were found among all variables (*ps* < 0.001). Sports training types can directly predict academic self-efficacy (β = 0.069, *t* = 3.155, *p* < 0.01), further confirming that sports training types can directly predict academic self-efficacy. Moreover, mindfulness, social anxiety, and academic procrastination play a chain mediating role between Artistic sports and academic self-efficacy.

**Discussion:**

These findings highlight the potential value of Artistic sports in enhancing academic self-efficacy and provide practical guidance for education policymakers, school administrators, teachers, parents, and students to promote adolescent academic and psychological health development. It is recommended to enhance the promotion and training of Artistic sports.

## 1 Introduction

In today’s society, academic self-efficacy has become a core topic in educational psychology and school education research. Academic self-efficacy is defined as learners’ judgments of their ability to successfully achieve learning goals (Elias and MacDonald, 2007). Students with high academic self-efficacy are more likely to set higher goals and exert more effort compared to those who doubt their academic abilities (Bandura, 1982, 1993). Academic self-efficacy not only influences learning motivation and academic achievement but is also closely related to students’ psychological well-being (Alivernini and Lucidi, 2011; Cobo-Rendón et al., 2020). Research findings indicate that current students often face challenges such as social anxiety and academic procrastination in their academic development, which may undermine their academic self-efficacy (NikMehr et al., 2021; Arias-Chávez et al., 2020). Therefore, exploring ways to enhance academic self-efficacy is crucial for promoting students’ overall development.

Sports activities have been found to be beneficial factors in enhancing self-efficacy (Latino et al., 2023; Ouyang et al., 2020). In August 2019, the “Outline of Building a Sports Power” highlighted the critical role of national fitness in promoting public health and building a sports power, emphasizing the importance of “national fitness” in the overall development strategy of the country. In October 2020, the General Office of the CPC Central Committee and the State Council jointly issued opinions on comprehensively strengthening and improving school sports and aesthetic education in the new era, underscoring the importance of enhancing school sports and aesthetic education.

”Artistic sports,” as a combination of art and sports, have unique charm and characteristics. Artistic sports are physical activities primarily aimed at entertaining the body and mind, relieving stress, and regulating emotions. This form not only has aesthetic value but also allows participants to personally experience joy and fun. It combines fitness, entertainment, and aesthetics, promoting physical health, stress relief, emotional regulation, etc., enhancing people’s physical fitness and health levels while providing a pleasant mind-body experience (Hu and Tian, 2006a; Liao and Li, 2024).

In recent years, more research has focused on the positive impact of artistic sports on students’ academic self-efficacy (Kpame and Richard, 2020; Moore et al., 2023; Su, 2024; Thieser et al., 2021). Artistic sports activities not only promote students’ physical and mental health development but also play an important role in cultivating mindfulness, reducing academic procrastination, social anxiety (Chen et al., 2024; Moratelli et al., 2023; Siregar et al., 2023; Khanbeiki, 2024). Additionally, mindfulness as an internal psychological resource helps students cope with stress and challenges, improve social anxiety and academic procrastination, and enhance academic self-efficacy (Dones et al., 2024; Li et al., 2023; Rad et al., 2023; Sharma and Kumra, 2022).

This study aims to explore the impact of artistic sports on college students’ academic self-efficacy, as well as investigate the mediating roles of mindfulness, social anxiety, and academic procrastination. By constructing a chain mediation model, the study delves into understanding the mechanisms through which artistic sports influence students’ academic development. The results indicate that artistic sports training can enhance academic self-efficacy by boosting mindfulness, reducing social anxiety, and alleviating academic procrastination. These findings suggest that schools and relevant institutions should strengthen the promotion and implementation of artistic sports training. They should provide practical recommendations for school educators and mental health professionals to enhance the physical and mental well-being of adolescents, promote comprehensive student development, and boost academic self-efficacy.

## 2 Literature review

### 2.1 Artistic sports

Artistic sports, as a fusion of modern sports science and art, are physical activities that aim to achieve precise technical movements and outstanding artistic expression while meeting the needs for physical and mental harmony (Zhang, 2009). This type of sports activity is a product of the times, characterized by artistic recreation based on aesthetic consciousness and evaluated using multiple criteria such as technical movements and artistic effects (Hou, 2011a,2011b). Artistic sports encompass various types of sports including gymnastics, dance, synchronized swimming, diving, trampolining, and ice dancing. These disciplines not only enhance individuals’ physical coordination, balance, and fitness but also foster social skills and teamwork spirit (Hu and Tian, 2006b). Research indicates that artistic sports have a significant effect on improving adolescents’ physical coordination, balance, and fitness while also playing an important role in enhancing their artistic expression (Boraczyński et al., 2013; Dos Santos et al., 2021). Therefore, artistic sports are not just a form of physical exercise but also a form of artistic expression that enriches individuals’ spiritual world and enhances their quality of life. As an activity that combines sports and art, artistic sports have a positive impact on academic self-efficacy. Studies show that students participating in artistic sports demonstrate higher academic self-efficacy (Reishehrei et al., 2014; Latino et al., 2023; Ouyang et al., 2020). Furthermore, mindfulness practices within artistic sports can help alleviate students’ test anxiety, further improving learning efficiency and academic performance (Beder, 2024; Bower and Carroll, 2015; Miyata et al., 2020; Shapiro and Bartlett, 2018).

### 2.2 Mindfulness

Mindfulness is typically described as a state of consciousness where individuals attentively focus on their present experiences with an open and accepting attitude, without making immediate reactions or judgments (Bishop et al., 2004). In psychological research, mindfulness is seen as a cultivatable skill. Through meditation and other mindfulness practices, individuals can enhance their levels of mindfulness, enabling them to exhibit better adaptability and coping abilities when facing stress, emotional disturbances, and cognitive challenges (Hölzel et al., 2011). This state of awareness involves non-judgmental attention to current experiences, including thoughts, emotions, sensations, and the surrounding environment (Keng et al., 2011).

Research has shown that mindfulness practices have a significant impact on adolescents’ performance and psychological well-being. Mindfulness training helps students focus on the present moment, increase awareness and control over their abilities, reduce social anxiety and stress levels, enhance performance in high-pressure situations, boost confidence in learning abilities, and potentially improve academic performance and motivation (Antonio, 2023; Singh and Singh, 2022; Zhang et al., 2022). In the context of artistic sports, mindfulness not only enhances skills but also strengthens individuals’ academic self-efficacy, benefiting both academic and personal development in adolescents.

### 2.3 Social anxiety

Social Anxiety is characterized as a state of tension and unease in social situations due to the fear of negative evaluation, often accompanied by avoidance behaviors such as reducing the frequency of appearances in public settings and feeling uncomfortable or fearful in situations requiring social interaction (American Psychiatric Association, 2013; Leary, 2021; Schlenker and Leary, 1982; Watson and Friend, 1969). Social anxiety involves not only negative emotional responses but also self-consciousness and cognitive worries arising from unmet social role and behavioral expectations (Horenstein et al., 2019; Morrison and Heimberg, 2013). Existing research suggests that artistic sports and mindfulness may serve as protective factors against social anxiety. Firstly, sports activities provide adolescents with a non-threatening social environment, helping them gradually build social confidence, thereby reducing levels of social anxiety and enhancing their ability to cope with social situations (Luna et al., 2019; Tunç and Akandere, 2020). Mindfulness training aids students in recognizing and managing their social anxiety, thereby building stronger confidence and motivation in academics (Ataei et al., 2020; Hashemi et al., 2020). Both artistic sports and mindfulness training play significant roles in promoting students’ social and academic development in educational environments.

### 2.4 Academic procrastination

Academic procrastination refers to individuals delaying the completion of tasks in a learning context (Senécal et al., 1995), which can have negative effects on individuals. Research indicates that academic procrastination not only impacts students’ academic performance and exam outcomes (Wang, 2021; Yang et al., 2020) but may also reduce individuals’ subjective well-being and life satisfaction (Çelik and Odaci, 2022; Çıkrıkçı and Erzen, 2020), leading to self-blame, guilt, and other negative emotions that can adversely affect students’ mental health (Balkis and Duru, 2018; Choi, 2021). Guiding college students to reduce academic procrastination behavior is an important issue faced by higher education professionals. Some studies suggest that social anxiety is a risk factor for academic procrastination (Seo, 2024), while individual levels of mindfulness serve as protective factors against academic procrastination. Higher levels of mindfulness predict lower levels of academic procrastination under certain conditions (Eltayeb, 2021; Schutte and del Pozo de Bolger, 2020).

### 2.5 Academic self-efficacy

Academic self-efficacy is a core concept in Bandura’s self-efficacy theory and is an essential component of self-concept. It refers to an individual’s perceived belief in their ability to achieve established goals (Bandura, 1977). Academic self-efficacy determines the level of effort individuals put into their studies, their perseverance in overcoming challenges, and is a key factor influencing student engagement and academic performance. Individuals with higher academic self-efficacy are more likely to actively face learning challenges, maintain effort and perseverance, and have the confidence to overcome difficulties. Conversely, individuals with lower academic self-efficacy may feel discouraged, anxious, or inclined to give up when facing academic difficulties (Qin et al., 2024; Tang et al., 2022). By cultivating and enhancing academic self-efficacy, individuals can better cope with learning challenges, improve academic performance, and enhance their commitment and motivation toward learning tasks. Understanding how to enhance academic self-efficacy is crucial for individual success in academics. Research indicates that physical exercise and mindfulness have positive implications for academic self-efficacy (Latino et al., 2023; Sharma and Kumra, 2022; Su, 2024). On the other hand, social anxiety and academic procrastination, common challenges faced by students in campus life, have negative relationships with self-efficacy (Morin-Huapaya et al., 2023; Nikoogoftar and Hajkazemi, 2022). Therefore, this study focuses on university artistic sports training as the independent variable to explore its relationship with students’ academic self-efficacy.

## 3 Research methodology

### 3.1 Participants

This study primarily investigated the significance of artistic sports training in higher education teaching by utilizing principles of convenience sampling and random sampling to conduct a questionnaire survey among students from selected universities in Gansu Province. The questionnaires were filled out online via the Questionnaire Star platform, and prior to the survey, all students voluntarily read and signed informed consent forms. In total, 1,976 questionnaires were collected, with an average age of 19.650 ± 2.445. There were 263 male participants (13.310%) and 1,713 female participants (86.690%). Among them, 433 students (21.913%) engaged in basic physical training, while 1,543 students (78.087%) participated in artistic sports training. For specific demographic information, please refer to Table 1.

**TABLE 1:** General demographic variables (*N* = 1976).

		*N*	Percentage
Gender	Male	263	13.31%
	Female	1713	86.69%
Place of origin town	Urban	355	17.97%
	Rural	1621	82.03%
Grade	Freshman	581	29.40%
	Sophomore	1320	66.80%
	Junior	68	3.44%
	Senior	3	0.15%
	Fifth year of college	2	0.10%
	Third year Master’s degree	2	0.10%
Family structure	Only child	224	11.34%
	Non-only child	1752	88.66%
Type of sport basic	Basic sports	433	21.91%
	Arts Sports	1543	78.09%

### 3.2 Research instruments

#### 3.2.1 General demographic questionnaire

A self-designed survey questionnaire consisting of basic demographic information such as gender, age, grade, place of origin, family structure, and additional inquiries regarding the participants’ engagement in art and sports training.

#### 3.2.2 Academic self-efficacy scale

The academic self-efficacy scale used in this study was developed by Pintrich (1991) and adapted by Xiong (2020) to assess individuals’ self-efficacy in academic domains tailored to local student characteristics. This scale comprises two dimensions - ability self-efficacy and behavioral self-efficacy - with 11 items each, totaling 22 items, where items 14, 16, 17, and 20 are reverse-coded. The internal consistency coefficient α for this scale was found to be 0.954 in this study, indicating high reliability.

#### 3.2.3 Social anxiety scale

The social anxiety scale utilized in this study was developed by Leary (1983) and published in the “Handbook of Psychological Assessment Scales” without modification to measure participants’ levels of social anxiety. Comprising two dimensions - tension and relaxation - the scale consists of 15 items including 4 reverse-scored items rated on a Likert scale ranging from “1” = “Not at all true” to “5” = “Very true.” The internal consistency coefficient for this scale was found to be 0.782.

#### 3.2.4 Academic procrastination scale

This study employed the academic procrastination scale developed by Solomon and Rothblum (1984), revised by Guan (2006), to evaluate students’ tendencies in academic procrastination across six aspects: writing term papers, reviewing coursework material, completing weekly assignments, managing academic tasks, engaging in activities, and general behavior on campus - totaling 18 items scored on a Likert-type scale from “Never” to “Always.” A higher score indicates greater levels of academic procrastination. The scale demonstrated good reliability with an internal consistency coefficient of 0.985.

#### 3.2.5 Five factor mindfulness questionnaire

The mindfulness questionnaire utilized in this study was based on the five-factor mindfulness model developed by Baer et al. (2006) and adapted into a concise Chinese version by Hou et al. (2014) known as the Five Facet Mindfulness Questionnaire (FFMQ). It includes 20 items across five dimensions (observing, describing, acting with awareness, non-judging of inner experience, non-reactivity), rated on a Likert scale from 1 (“Never true”) to 5 (“Always true”). Higher total scores indicate greater levels of mindfulness. The internal consistency coefficient α for this scale was found to be 0.975, demonstrating strong reliability.

### 3.3 Statistical analysis

This study utilized SPSS 26.0 and the PROCESS plugin for data analysis. Specific analyses included descriptive statistics, differences analysis through independent sample *t*-tests, examination of variable correlations using Pearson correlation analysis, mediation analysis and effect testing through regression analysis using the PROCESS tool. A significance level of *p* < 0.05 was considered to reach statistical significance. Significance testing involves examining hypotheses about the population and is based on the principle of “improbability of rare events” to determine whether to accept or reject a hypothesis. The significance level is a crucial concept in hypothesis testing, representing the probability or risk of rejecting the null hypothesis when it is actually true. It indicates the probability value considered as a rare event and needs to be determined before each statistical test, commonly set at α = 0.05 or α = 0.01. This means that when deciding to accept the null hypothesis, there is a 95 or 99% chance of being correct. In this study, a significance level of α = 0.05 was used throughout the analyses.

## 4 Results

### 4.1 Common method bias

In this study, all data on variables were gathered through self-reported questionnaires by the participants, potentially introducing common method bias that could impact the research outcomes. To address this concern, Harman’s single-factor test was conducted to assess the presence of common method bias in the study. The results revealed that the first factor without rotation explained 28.317% of the variance, which is below the critical threshold of 40%, indicating no significant common method bias.

### 4.2 Basic demographic information

The collected questionnaire data was meticulously organized, and specific demographic information is presented in Table 1. Among the 1,976 participants surveyed in this study, there were 263 males (13.31%) and 1,713 females (86.69%); 355 individuals from urban areas (17.97%) and 1,621 individuals from rural areas (82.03%); distribution across academic years included: freshmen 581 (29.40%), sophomores 1,320 (66.80%), juniors 68 (3.44%), seniors 3 (0.15%), fifth-year students 2 (0.10%), third-year graduate students 2 (0.10%), with no data available for first and second-year graduate students; there were 224 only children (11.34%) and 1,752 non-only children (88.66%); 433 participants engaged in basic sports training (21.91%), while 1,543 participants participated in Artistic sports training (78.09%).

### 4.3 Comparative analysis

Independent Samples *t*-Test Analysis of Gender, Only Child Status, and Place of Origin Differences in Mindfulness, Academic Self-Efficacy, Social Anxiety, and Academic Procrastination (see Table 2). The results indicate that there were no significant differences in mindfulness and academic self-efficacy based on gender (*t* = −0.584, *p* > 0.05; *t* = −0.229, *p* > 0.05). However, significant disparities were observed in social anxiety and academic procrastination across genders (*t* = −4.889, *p* < 0.001; *t* = −2.255, *p* < 0.05). There were no significant variances in the variables related to only child status and place of origin (*ps* > 0.05). Notably, significant differences were found among the types of sports training participated in by individuals (*ps* < 0.05), with participants engaging in Artistic sports training demonstrating higher levels of mindfulness and self-efficacy while reporting lower levels of social anxiety and academic procrastination.

**TABLE 2:** Basic demographic disparities across variables (*N* = 1976).

	*N*	Mindfulness (*M* ± + )	Social anxiety (M ± SD)	Academic procrastination (M ± SD)	Academic self-efficacy (M ± SD)
Male	263	58.551 ± 20.032	42.852 ± 7.632	47.327 ± 17.764	67.726 ± 13.781
Female	1713	57.896 ± 16.427	45.304 ± 7.169	48.909 ± 13.760	67.915 ± 12.208
*t*		−0.584	−4.889***	−2.255*	−0.229
Only child	224	57.866 ± 19.306	45.348 ± 8.731	47.388 ± 16.750	68.125 ± 13.383
Non-only child	1752	57.998 ± 16.627	44.930 ± 7.072	48.715 ± 14.045	67.860 ± 12.301
*t*		−0.110	0.810	−1.301	0.301
Urban	355	57.541 ± 19.486	45.279 ± 7.888	48.996 ± 16.001	67.637 ± 13.638
Rural	1621	58.080 ± 16.343	44.911 ± 7.138	48.477 ± 14.003	67.945 ± 12.148
*t*		−0.543	0.862	0.581	−0.424
Basic sports	433	55.515 ± 16.755	45.688 ± 7.064	50.060 ± 13.992	65.758 ± 12.396
Arts Sports	1543	58.676 ± 16.941	44.778 ± 7.327	48.145 ± 14.462	68.488 ± 12.372
*t*		−3.461***	2.351*	2.498*	−4.052***

*p < 0.05,

***p < 0.001; “p” is the probability, reflecting the probability of an event.

### 4.4 Correlation analysis

Pearson correlation analysis was conducted to explore the relationships between mindfulness, social anxiety, academic procrastination, and academic self-efficacy (see Table 3). The results indicated a significant negative correlation between mindfulness and both social anxiety (*r* = −0.179, *p* < 0.001) and academic procrastination (*r* = −0.157, *p* < 0.001), along with a significant positive correlation with academic self-efficacy (*r* = 0.182, *p* < 0.001). Social anxiety and academic procrastination were positively correlated (*r* = 0.483, *p* < 0.001) while demonstrating a negative correlation with academic self-efficacy (*r* = −0.179, *p* < 0.001). Furthermore, academic procrastination exhibited a negative correlation with academic self-efficacy (*r* = −0.185, *p* < 0.001).

**TABLE 3:** Correlation test of variables (*N* = 1976).

	1. Mindfulness	2. Social anxiety	3. Academic procrastination
2. Social anxiety	−0.179***		
3. Academic procrastination	−0.157***	0.483***	
4. Academic self-efficacy	0.182***	−0.179***	−0.185***

***p < 0.001; “p” is the probability, reflecting the probability of an event.

### 4.5 Mediation analysis

Utilizing Hayes’ SPSS macro-PROCESS with model 6 and 5000 bootstrap samples to calculate a 95% confidence interval, the mediating roles of mindfulness, social anxiety, and academic procrastination in the relationship between types of sports training and academic self-efficacy were examined.

As indicated in Table 4, the majority of pathways were found to be statistically significant. Types of sports training were able to significantly predict academic self-efficacy (β = 0.069, *t* = 3.155, *p* < 0.01). Moreover, mindfulness, social anxiety, and academic procrastination were all able to significantly predict academic self-efficacy (β = 0.142, *t* = 6.411, *p* < 0.001; β = −0.095, *t* = −3.803, *p* < 0.001; β = −0.113, *t* = −4.523, *p* < 0.001). Types of sports training did not significantly predict academic procrastination and social anxiety (β = −0.025, *t* = −1.284, *p* < 0.05; β = −0.038, *t* = −1.720, *p* < 0.05). However, mindfulness and social anxiety were able to significantly predict academic procrastination (β = −0.071, *t* = −3.558, *p* < 0.001; β = 0.469, *t* = 23.480, *p* < 0.001). Mindfulness was also found to predict social anxiety (β = −0.176, *t* = −7.935, *p* < 0.001). Additionally, types of sports training were able to predict levels of mindfulness (β = 0.077, *t* = 3.439, *p* < 0.001).

**TABLE 4:** Mediation analysis of mindfulness, social anxiety, and academic procrastination (*N* = 1976).

Outcome variable	Independent variable	β	*t*	*p*
AS	STT	0.069	3.155	0.002
	FFM	0.142	6.411	0.000
	SA	−0.095	−3.803	<0.001
	AP	−0.113	−4.523	0.000
AP	STT	−0.025	−1.284	0.199
	FFM	−0.071	−3.558	<0.001
	SA	0.469	23.480	0.000
SA	STT	−0.038	−1.720	0.086
	FFM	−0.176	−7.935	0.000
FFM	STT	0.077	3.439	<0.001

STT, sports training types; FFM, five factor mindfulness; SA, social anxiety; AP, academic procrastination; AS, academic self-efficacy.

**FIGURE 1:**
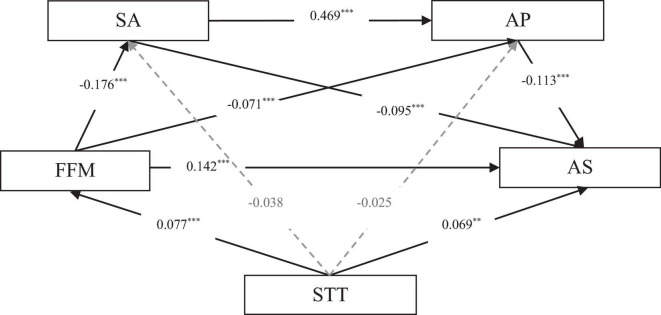
The Mediation model of mindfulness, social anxiety, and academic procrastination in the relationship between types of sports training and academic self-efficacy. STT, sports training types; FFM, five factor mindfulness; SA, social anxiety; AP, academic procrastination; AS, academic self-efficacy. **p < 0.01, ***p < 0.001; “p” is the probability, reflecting the probability of an event.

The diagram of the serial mediation model is shown in Figure 1:

As shown in Table 5, the direct effect of types of sports training on academic self-efficacy is 0.0688, with a confidence interval of [0.0260, 0.1116]. The total indirect effect is 0.0221, with a confidence interval of [0.0109, 0.0350]. The total effect is 0.0909, with a confidence interval of [0.0470, 0.1349], where the confidence interval does not include 0, indicating significance. The mediating effect through mindfulness is found to be 0.0110, with a confidence interval of [0.0041, 0.0193], demonstrating a significant mediating role and supporting the mediation hypothesis.

**TABLE 5:** Testing the mediation effects of mindfulness, social anxiety, and academic procrastination (*N* = 1976).

	Effect	Bootstrap SE	Bootstrap 95% CI	
			**Low**	**High**
Direct effects	0.0688	0.0218	0.0260	0.1116
STT→FFM→AS	0.0110	0.0039	0.0041	0.0193
STT→SA→AS	0.0036	0.0024	−0.0003	0.0091
STT→AP→AS	0.0029	0.0025	−0.0014	0.0081
STT→FFM→SA→AS	0.0013	0.0006	0.0004	0.0027
STT→FFM→AP→AS	0.0006	0.0004	0.0001	0.0015
STT→SA→AP→AS	0.0020	0.0014	−0.0002	0.0051
STT→FFM→SA→AP→AS	0.0007	0.0003	0.0002	0.0014
Total indirect effects	0.0221	0.0062	0.0109	0.0350
Total effects	0.0909	0.0224	0.0470	0.1349

STT, sports training types; FFM, five factor mindfulness; SA, social anxiety; AP, academic procrastination; AS, academic self-efficacy.

The mediating effect through social anxiety is found to be 0.0036, with a confidence interval of [−0.0003, 0.0091], which does not reach significance, indicating that the mediation hypothesis is not supported. Similarly, the mediating effect through academic procrastination is 0.0029, with a confidence interval of [−0.0014, 0.0081], suggesting that the mediation hypothesis is not supported.

On the other hand, when both mindfulness and social anxiety are considered as mediating variables simultaneously, the effect is 0.0013, with a confidence interval of [0.0004, 0.0027]. Analyzing the combined mediation of mindfulness and academic procrastination yields an effect of 0.0006, with a confidence interval of [0.0001, 0.0015]. In both cases, the confidence intervals do not include 0, indicating significant mediating effects and supporting the conclusion that mediation exists in these models.

The combined mediating effect of social anxiety and academic procrastination is found to be 0.0020, with a confidence interval of [−0.0002, 0.0051], where the confidence interval includes 0, indicating that the mediating effect is not significant and the mediation hypothesis is not supported. However, when considering mindfulness, social anxiety, and academic procrastination simultaneously as mediating variables, the effect is calculated to be 0.0007, with a confidence interval of [0.0002, 0.0014]. In this case, the confidence interval does not include 0, suggesting a significant mediating effect and supporting the conclusion that serial mediation exists.

Therefore, types of sports training can influence academic self-efficacy through the mediating roles of mindfulness, social anxiety, and academic procrastination.

## 5 Discussion

This study explores the impact of artistic sports and traditional sports training on college students’ academic self-efficacy, while investigating the mediating roles of mindfulness, social anxiety, and academic procrastination. Artistic sports, through their unique creative and expressive activities, cultivate students’ sense of engagement and initiative, thereby enhancing their social skills and overall qualities.

Firstly, in the differential analysis for this study, we found that there were no significant gender differences in mindfulness and academic self-efficacy. However, significant differences were observed in social anxiety and academic procrastination, with females reporting higher levels of social anxiety and academic procrastination. This finding is consistent with previous research results (Asher et al., 2017; Ghosh and Roy, 2017), indicating that gender differences in social anxiety and academic procrastination may require more attention and intervention. The study also found that students engaged in artistic sports scored higher in mindfulness and self-efficacy, while scoring lower in social anxiety and academic procrastination. This aligns with previous research results (Su, 2024; Chen et al., 2024; Moratelli et al., 2023; Siregar et al., 2023; Khanbeiki, 2024), highlighting the positive impact of artistic sports on adolescents.

The results of Pearson correlation analysis indicated significant relationships among all variables. The chain mediation model analysis conducted using PROCESS revealed that artistic sports training positively predicts academic self-efficacy and exerts a positive influence on academic self-efficacy through enhancing mindfulness, reducing social anxiety, and academic procrastination. Mindfulness, social anxiety, and academic procrastination mediate the relationship between type of sports training and academic self-efficacy. Notably, in the model, the direct predictive effect of sports training type on social anxiety and academic procrastination was not significant but had a significant indirect predictive effect. Nevertheless, our hypothesis was supported by these findings. These results suggest that schools and relevant institutions should strengthen the promotion and implementation of artistic sports training to better enhance the physical and mental well-being of adolescents and promote academic development. Below are some specific recommendations proposed based on these findings.

### 5.1 Suggestions

From the perspective of the education management department: Education policymakers should promote in-depth research on the connection between artistic sports and academic self-efficacy and use these findings to formulate comprehensive educational policies. This includes providing necessary financial and facility support to schools to ensure the smooth implementation of artistic sports programs. It is also essential to establish a robust system for professional development for teachers, enhancing their expertise in artistic sports teaching through regular training and workshops. Furthermore, relevant departments should develop effective monitoring and evaluation mechanisms to ensure policy implementation, allowing for timely adjustments based on evaluation results to uphold the quality and effectiveness of artistic sports education.

From the school’s perspective: Schools should consider artistic sports as a crucial component in fostering students’ holistic development. By innovatively combining art and physical education in curriculum design, schools can offer a diverse range of sports activities for students to choose from. Schools need to prioritize faculty development by improving teachers’ expertise in artistic sports teaching through professional training. By organizing various traditional sports and art events, schools can create a positive campus culture. Additionally, schools should establish comprehensive mental health support services to provide necessary counseling and guidance for students, helping them cope with social anxiety and academic pressure.

From the perspective of educators (teachers): Teachers play a crucial role in guiding students’ improvement in academic self-efficacy. To enhance student engagement and interest in learning, teachers should continuously explore and implement innovative teaching methods. They need to address individualized student needs by providing differentiated instructional support to help each student reach their full potential. In the collaboration between home and school, teachers should maintain close communication with parents to collectively focus on students’ academic progress and personal development, promoting a positive home-school partnership. Continuous professional development for teachers is equally vital; they must continually learn new educational concepts and teaching techniques to adapt to new trends in education.

From the parents’ perspective: Parents play a vital role in their children’s growth process. They should actively encourage children to participate in physical and artistic activities while recognizing the positive impact of these activities on their children’s physical and mental health as well as social skills development. Parents need to create a loving and supportive family environment that helps children build confidence and overcome social anxiety. When children face academic pressures and challenges, parents should provide appropriate supervision and guidance, assisting them in developing reasonable study plans and cultivating good study habits. Parents should actively engage in school activities, communicate with teachers regularly, and collectively focus on their children’s academic progress and mental health.

From the students’ perspective: Students are the protagonists of their own development. As modern-day university students, they should actively participate in physical and artistic courses provided by schools to enhance their physical fitness, mental qualities, and social skills. Students need to learn time management and self-monitoring skills to improve study efficiency while reducing academic procrastination. When facing academic difficulties or psychological stress, students should bravely seek help from teachers, parents or professional counselors. They should also cultivate healthy lifestyle habits including balanced dieting, adequate sleep, regular exercise to maintain good physical and mental health as a solid foundation for academic and personal growth.

### 5.2 Shortcomings and prospects

This study provides valuable insights into the impact of artistic sports on college students’ academic self-efficacy and its influencing mechanisms, which can help professionals, students, and relevant institutions better understand the advantages of artistic sports and organize sports activities more effectively. However, there are some limitations that need to be improved and expanded in future research. Firstly, this study utilized a cross-sectional research design, limiting our ability to infer causality. Future research could employ longitudinal research designs to better understand the developmental changes and causal relationships between artistic sports, mindfulness, social anxiety, academic procrastination, and academic self-efficacy. Secondly, although this study had a large sample size, the sample was limited to university students from a specific region and may not represent all university student populations. Future studies should consider expanding the sample range to include students from different regions and cultural backgrounds to enhance the universality and generalizability of the research results. Additionally, there was a significant disparity in the gender ratio and the distribution of types of sports training in this study, which may have influenced the results to some extent. In future studies, attention should be paid to balancing the proportion of different categories as much as possible. Lastly, this study primarily focused on the impact of artistic sports on academic self-efficacy, while the effects of artistic sports on other psychological variables (such as emotional regulation, creativity, physical health, etc.) have not been deeply explored. Future research could expand its focus to these areas to comprehensively assess the holistic benefits of artistic sports.

## 6 Conclusion

This study reveals the positive role of artistic sports in enhancing college students’ academic self-efficacy. Engaging in artistic sports training can effectively boost students’ self-efficacy and improve their academic self-efficacy by enhancing mindfulness, reducing social anxiety, and academic procrastination. The findings emphasize the importance of strengthening artistic sports activities in educational practice. Schools and relevant institutions should increase the promotion and implementation of artistic sports training to support the holistic development of students’ physical and mental well-being.

## Data Availability

The raw data supporting the conclusions of this article will be made available by the authors, without undue reservation.
